# Is It Really SUMP Syndrome? A Case Report

**DOI:** 10.7759/cureus.5837

**Published:** 2019-10-04

**Authors:** Mohamed S Suliman, Monider M Singh, Kamran Zaheer, Saad Ullah Malik, Ahmad Abu-Hashyeh

**Affiliations:** 1 Internal Medicine, Marshall University, Joan C Edwards School of Medicine, Huntington, USA; 2 Internal Medicine, Abrazo Community Health Network, Glendale, USA; 3 Hematology and Oncology, University of Arizona, Tucson, USA

**Keywords:** sump syndrome, side-to-side choledochoduodenosotomy, pneumobilia, pancreatitis, endoscopic retrograde cholangiopancreatography

## Abstract

Sump syndrome is a rare, long-term complication with a prevalence ranging from 0% to 9.6% in patients with a history of side-to-side choledochoduodenostomy. Choledochoduodenostomy was originally performed to achieve drainage of the common bile duct in high-risk patients with low morbidity, which was commonly done in the pre-endoscopic retrograde cholangiopancreatography era. “Sump” comes from the segment of the common bile duct between the anastomosis and the ampulla of Vater, which acts as a stagnant reservoir for debris, stones, and static bile. This predisposes patients to changes in the biliary tree with signs and symptoms in relation to that area. If left untreated, cholangitis, pancreatitis, hepatic abscesses, and secondary biliary cirrhosis can develop. Here, we have a case of a 77-year-old male with a history significant for choledochoduodenostomy, who presented with the clinical signs and symptoms of pancreatitis, choledocholithiasis, and urinary tract infection. Computed tomography (CT) scan findings revealed choledocholithiasis and an enlarged common bile duct with smaller adjacent calculi along with pneumobilia consistent with sump syndrome. The patient’s clinical status improved without invasive measures being taken, i.e. endoscopic retrograde cholangiopancreatography. He was subsequently discharged home after improving clinically and no invasive measures were pursued.

## Introduction

Sump syndrome or blind sac syndrome is a rare, long-term complication in patients with a history of a side-to-side choledochoduodenostomy (CDD) [1-3]. Side-to-side CDD was originally performed to achieve decompression of the biliary ducts, typically the common bile duct (CBD), with low morbidity in high-risk patients, which was done in the era before ERCP (pre-ERCP) [1,3-4]. Most surgeons consider it the last resort in elderly patients with abnormally wide ducts [3]. Some indications for CDD include large impacted stones; choledocholithiasis with stricture; recurrent stones; and CBD with stricture and cholecystoduodenal fistula [5-6]. Potential complications from CDD are wound complication, cholangitis, biliary fistula, residual stone/recurrent stone, alkaline reflux gastritis, sump syndrome, septicemia, pancreatitis, undefined abdominal pain, pneumonia, postoperative fever, bile leak, and anastomotic stenosis [1,5-7]. 

The term “sump” comes from the segment of the common bile duct between the anastomosis (biliary-enteric) and the Ampulla of Vater, which may act as a stagnant reservoir for debris, stones, and static bile, i.e. sump [1,4,8-10]. This predisposes the patient to inflammatory/infectious changes in the biliary tree, with signs and symptoms including intermittent pain and tenderness in the right upper quadrant (RUQ), fever, jaundice, chills, nausea, and vomiting [2,6-8,11]. On imaging studies, such as computed tomography (CT) or magnetic resonance cholangiopancreatography (MRCP), findings warranting ERCP are debris or stone(s) in the biliary ducts, i.e. common bile duct [1,4]. Suggestive findings and potential complications from sump syndrome include dilated bile or pancreatic ducts, changes due to pancreatitis, cholangitis, cirrhosis, or hepatic abscesses [1,4,8,10-11]. Additional findings of air in the biliary tree (pneumobilia) and debris-filled ducts should lead to adding sump syndrome as a differential of abdominal pain [1-2,8].

The primary therapeutic and diagnostic options are ERCP or percutaneous transhepatic cholangiography (PTC) with bile duct clearance and, in some cases, a redo of the anastomosis [8,11]. With that in mind, sump syndrome is a rare complication that can go under-reported [4]. We report a case of a 77-year-old male, with a past surgical history of choledochoduodenostomy status post (s/p) one year who presented with abdominal pain radiating to his flanks, hematuria, dysuria, fatigue, and recent fall, who was diagnosed with sump syndrome on noninvasive imaging studies.

## Case presentation

This is a case of a 77-year-old male who presented to our emergency room at a university hospital with abdominal pain radiating to his flanks bilaterally, more prominent on the left side over the past four days, hematuria and dysuria of one-day duration, fatigue, and a recent fall within the last couple of days. The patient reported decreased oral intake and unintentional weight loss of 58 lbs in the past three months. He denied fever, chills, shortness of breath, chest pain, diarrhea, constipation, and swelling in his extremities. His medical history was significant for non-obstructive coronary artery disease (CAD) with recent echocardiogram (echo) ejection fraction (EF) >55%, hypertension (HTN), hyperlipidemia (HLP), chronic pancreatitis, atrial fibrillation (AFib) on apixaban, sick sinus syndrome s/p dual-chamber pacemaker, gastroesophageal reflux disease (GERD), benign prostatic hyperplasia (BPH), and gout. The patient’s surgical history was remarkable for cardiac catheterization with the placement of two stents, pacemaker placement, laparoscopic cholecystectomy, and the year prior's esophagogastroduodenoscopy/endoscopic ultrasound (EGD/EUS) evaluation of the pancreatic head mass, which was nonpathological on fine-needle aspiration (FNA), endoscopic retrograde cholangiopancreatography (ERCP) for the evaluation of dilated biliary ducts, percutaneous internal/external biliary ductal stent placement for relieving the symptoms of pancreatitis at the time, exploratory laparotomy, partial colectomy, choledochoduodenostomy (anastomosis between the distal portion of common bile duct and duodenum was created) due to complications from improper drain placement, hernia repair, right carotid endarterectomy, and colonoscopy. On physical exam, left lower quadrant (LLQ) pain was elicited with palpation with no rebound tenderness; suprapubic tenderness was noted.

**Figure 1:**
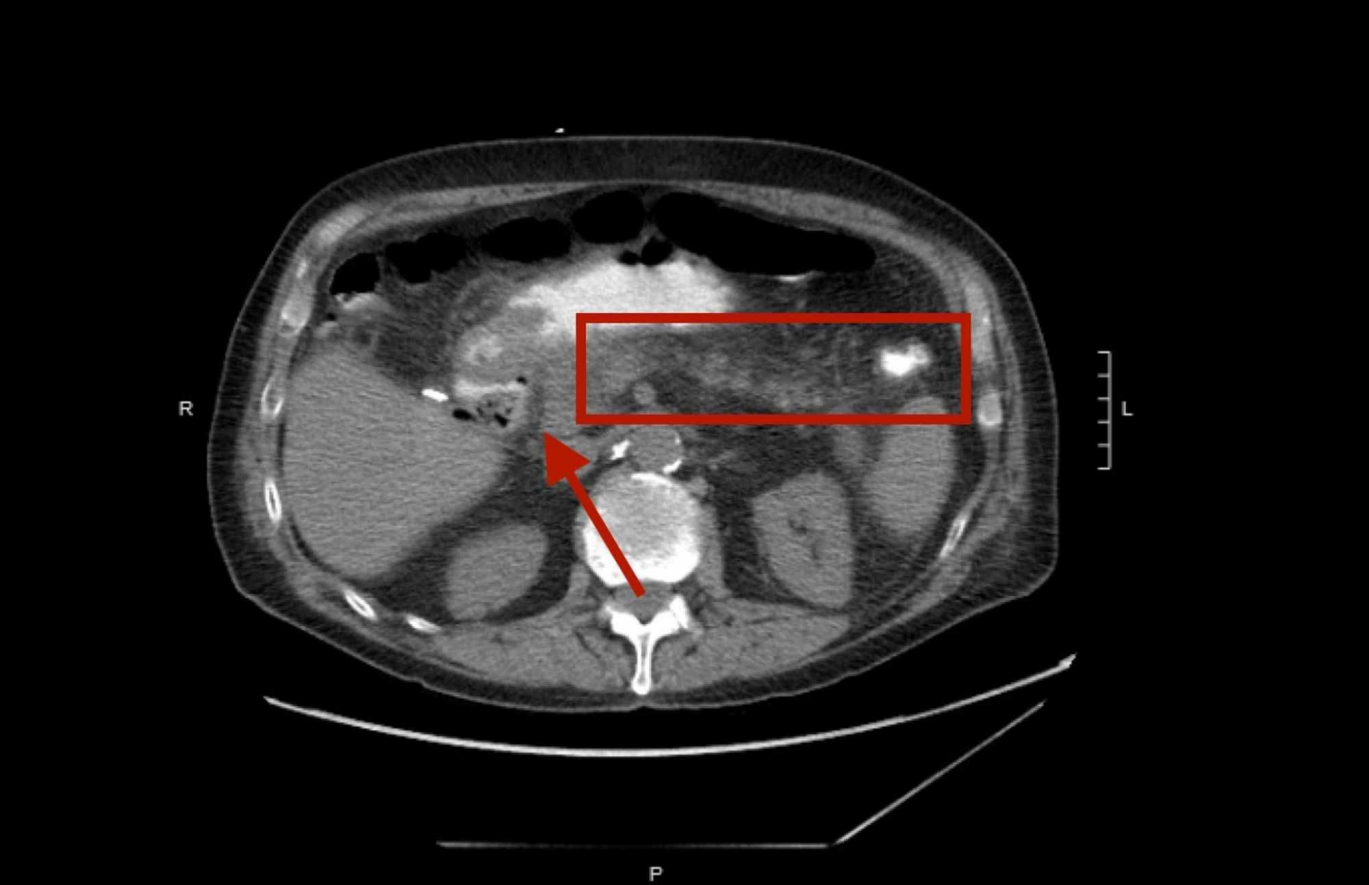
CT abdomen and pelvis without contrast

Given the patient's extensive surgical history, significant weight loss in the past three months, abdominal pain, hematuria, and dysuria, the patient was admitted for further inpatient evaluation. On admission, his vitals were a heart rate of 75, peripheral capillary oxygen saturation (SpO2) of 97%, temperature of 99.1°F, blood pressure of 158/97 mmHg, and respiratory rate of 16 breaths per minute. Initial lab studies revealed a white blood cell (WBC) count of 18.4 k/cmm, urinalysis (u/a) positive for occult blood, leukocyte esterase, with blood urea nitrogen (BUN)-to-creatinine ratio (BUN/Cr) at 21 mg/dL/1.86 mg/dL, albumin of 2.0, alkaline phosphatase of 122, aspartate transaminase/alanine transaminase (AST/ALT) of 37/25, total bilirubin of 0.6 mg/dL, calcium of 8.2 mg/dL, with correct ionized calcium of 0.99 mg/dL and lipase of 514. Initial imaging studies included an abdominal X-ray, which was remarkable for possible ileus, enteritis, or partial obstruction. Abdominal and retroperitoneal ultrasound revealed a 7.5-mm diameter calculus in the head of the pancreas and pancreatic duct dilated to a max diameter of 9 mm. Computed tomography (CT) scan of the abdomen and pelvis without contrast revealed s/p cholecystectomy, s/p choledochoduodenostomy, s/p partial right colectomy, choledocholithiasis, dilated CBD measuring 1.1 cm, in addition to smaller adjacent calculi along with pneumobilia and inflammatory changes of the pancreas, acute on chronic pancreatitis, and stated cholangitis should be considered along with findings consistent with sump syndrome (Figure 1).

The appendix was also found to be enlarged. Surgery and gastroenterology (GI) services were consulted. Surgery stated no need for emergent surgery for the enlarged appendix, and GI held off on proposed ERCP until further imaging results came back to definitively diagnose the patient's cause of abdominal pain.

The patient was subsequently started on Zosyn (piperacillin/tazobactam) for possible urinary tract infection (UTI) versus cholangitis, intravenous fluids (IVF) for prevention of acute kidney injury (AKI), intravenous (IV) Tylenol for controlling abdominal pain, pancreatic enzymes for digestive support, and pancreatic rest. GI continued to follow up for any deteriorating symptoms and signs. Within two days of hospital admission, his clinical status was improving with WBC counts trending down and the ability to eat solids as tolerated. The patient was discharged after a four-day hospital stay to home health and family care. The discharged diagnosis was confirmed to be calculus of bile duct w/o cholangitis or cholecystitis w/o obstruction.

## Discussion

Choledochoduodenostomy (CDD) was a common surgical procedure used in the pre-ERCP era for benign biliary tract diseases [1]. Currently, ERCP has taken the place of CDD; the complications from CDD continue to appear in the elderly population, leading to difficulties in diagnosis for current physicians [2]. Side-to-side CDD is an established procedure for the drainage of the CBD and is usually performed in the setting of multiple calculi or biliary sludge in the common bile duct or dilated (>15 mm) ducts [6]. An anastomosis is created between the bile duct, i.e. distal or proximal portion common bile duct and the duodenum [12-13]. The prevalence of sump syndrome remains quite low, ranging from 0% to 9.6% [14]. Sump syndrome has remained largely unrecognized by surgeons and physicians because of its relative infrequency and the lack of long-term follow-up studies after surgery [14]. Even with a low prevalence, if sump syndrome goes untreated, it can lead to a host of complications calling for physicians and surgeons to have more awareness of this syndrome.

Sump syndrome occurs due to bile stasis and reflux of duodenal contents in the portion of the common bile that is anastomosed with the duodenum resulting in a pool, allowing bacterial overgrowth and complications to arise [2,15]. Surgeons are trained to make this anastomosis widely patent so such a complication does not occur, but cases tend to sporadically appear from time to time [4,15]. Some factors that contribute are an inadequate stomal size and an unfavorable anastomotic configuration [15].

This entity can present with a variety of signs and symptoms in relation to the area where debris or bile is pooled. Diagnosis can be challenging, but, typically, abdominal radiographs tend to show air in the biliary tree (pneumobilia), calcifications in the right upper quadrant, and debris-filled dilated biliary ducts are suggestive. Ultrasound may show pneumobilia, biliary duct dilatation, biliary stones, changes of cholangitis, pancreatitis, pancreatic duct dilation, and liver abscess. CT tends to show prior surgical changes, debris, stones in the distal CBD, and enhancement of duct walls [1,8]. When the clinical picture is clear, the primary treatment and management of sump syndrome is endoscopic sphincterotomy and if that fails, ﻿many surgeons choose to undertake a Roux-en-Y choledochojejunostomy or hepaticojejunostomy [4,14-15].

In our case, the patients' imaging findings were suggestive for sump syndrome along with the clinical picture. The syndrome was not pursued due to the patient’s extensive surgical history and GI service, feeling ERCP inappropriate at the time, as one indication is to have a dilated CBD size of at least 1.5 cm and in our case, the patient’s CBD was only dilated to 1.1 cm [5-6]. Instead, a wait-and-watch approach was adopted, and the patient improved throughout the hospital course. If the patient continued to have abdominal symptoms with more definitive features of sump syndrome, the option of ERCP would have been pursued. A conservative strategy was taken on the probable basis that not all indications were met and the rarity of the complication happening relatively quickly. The average time frame for sump syndrome to appear ranges from six and 11 years [7,14]. As reported, if picked up early on radiographs and imaging studies, suggestive findings of sump syndrome can seem definitive, but one has to not only thoroughly consider all indications and the overall clinical picture before taking the next step in invasive treatment but also to consider the possibility to treat conservatively as was the case here.

## Conclusions

Due to its rarity, it is important to include sump syndrome in the differential diagnosis for patients who present with the signs and symptoms of cholangitis, pancreatitis, and abdominal pain, with a history of cholecystectomy and CDD in the post-ERCP era. The diagnosis of sump syndrome can be challenging because no one characteristic, be it clinical or laboratory finding, is specific. If clinical findings and imaging suggest sump syndrome, early conservative therapy can result in resolution as was seen in our case. There seems to be a consensus on when ERCP is indicated and that seems to be a point of focus in previous cases. Having a clear clinical picture and a high degree of suspicion is the most important element for diagnosing, treating, and preventing the complications of sump syndrome.
